# Machine Learning and Deep Learning for Earthquake Monitoring: A Systematic Review of Distributed Acoustic Sensing Applications

**DOI:** 10.3390/s26175542

**Published:** 2026-08-31

**Authors:** Nimra Iqbal, Izzatdin Bin Abdul Aziz, Halimaton Saadiah Bt Hakimi, Muhammad Faisal Raza, Alidu Rashid

**Affiliations:** 1 Department of Computer & Information Sciences, Universiti Teknologi Petronas (UTP), Seri Iskandar 32610, Perak Darul Ridzuan, Malaysia; izzatdin@utp.edu.my (I.B.A.A.); halimaton.hakimi@utp.edu.my (H.S.B.H.); 2 Department of Electrical & Electronic Engineering, Universiti Teknologi Petronas (UTP), Seri Iskandar 32610, Perak Darul Ridzuan, Malaysia; muhammad_24009644@utp.edu.my; 3 Department of Petrolleum Engineering, Universiti Teknologi Petronas (UTP), Seri Iskandar 32610, Perak Darul Ridzuan, Malaysia; alidu.rashid@utp.edu.my

**Keywords:** machine learning, deep learning, artificial intelligence, distributed acoustic sensing (DAS), seismic event detection, systematic review

## Abstract

Earthquakes remain among the most destructive natural hazards, necessitating reliable monitoring and early warning systems for effective risk mitigation. Recent advances in machine learning (ML) and deep learning (DL) have significantly improved seismic signal analysis, enabling more accurate event detection, phase picking, classification, and magnitude estimation. This study presents a systematic review of ML- and DL-based approaches for earthquake monitoring, with particular emphasis on Distributed Acoustic Sensing (DAS) as an emerging technology for high-resolution, real-time seismic observation. Following the PRISMA 2020 guidelines, a systematic literature search was conducted across Scopus, Web of Science, IEEE Xplore, and Google Scholar, yielding 252,223 initial records. After applying the predefined publication period, removing duplicate records, conducting relevance screening, and performing eligibility assessment, 138 peer-reviewed studies published between 2021 and 2025 were retained for detailed analysis and synthesis. The review reveals a significant transition from conventional signal-processing techniques to advanced artificial intelligence-based approaches, including Convolutional Neural Networks (CNNs), Long Short-Term Memory (LSTM) networks, Bidirectional Long Short-Term Memory (BiLSTM) networks, Transformer-based architectures, hybrid models, and Bayesian learning methods for uncertainty quantification. The findings further demonstrate that Distributed Acoustic Sensing (DAS) has emerged as a transformative sensing technology because of its dense spatial coverage, high spatial resolution, and continuous monitoring capability. However, several challenges remain, including the lack of standardized datasets, limited model generalization across diverse geological settings, insufficient model interpretability, high computational complexity, and the limited integration of uncertainty-aware approaches for real-time seismic monitoring. This review identifies these critical research gaps and highlights promising future research directions, including multimodal data fusion, interpretable artificial intelligence, physics-informed learning, self-supervised learning, and robust uncertainty quantification for next-generation intelligent seismic monitoring systems. Unlike previous review studies that primarily focus on individual machine learning techniques or conventional seismic monitoring, this review provides a comprehensive and systematic synthesis of recent advances in machine learning, deep learning, and Distributed Acoustic Sensing (DAS), identifies current research gaps, and offers practical recommendations to guide future research on intelligent earthquake monitoring systems.

## 1. Introduction

Recent advances in seismic sensing technologies, particularly broadband seismometers and Distributed Acoustic Sensing (DAS) systems based on fiber-optic cables, have transformed earthquake monitoring and subsurface characterization [1]. These sensing technologies provide continuous, high-density measurements with unprecedented spatial and temporal resolution, enabling detailed observation of seismic wave propagation, microseismic activity, and transient deformation processes in both natural and engineered environments. However, these capabilities have also led to a dramatic increase in the volume and complexity of seismic data, making manual interpretation and conventional rule-based signal processing methods increasingly inadequate for timely and reliable analysis [2].

Traditional approaches to seismic event detection and phase picking typically rely on threshold-based algorithms, handcrafted features, or statistical models. Although effective under controlled conditions, these methods often exhibit limited robustness in the presence of strong background noise, sensor variability, changing coupling conditions, and non-stationary seismic signals [3]. These challenges become even more pronounced in DAS-based monitoring, where dense spatial sampling generates highly correlated multichannel time-series data that are substantially larger and more complex than those acquired from conventional seismic networks.

To address these limitations, machine learning (ML) and deep learning (DL) have emerged as transformative technologies for seismic data processing. These approaches automatically learn hierarchical feature representations directly from raw waveforms, spectrograms, or multicomponent seismic signals while maintaining scalability for continuously growing datasets [4]. Deep learning has demonstrated remarkable success in a wide range of seismic interpretation tasks, including seismic facies classification, geological feature extraction, event detection, phase picking, and subsurface characterization. Supervised deep learning frameworks have significantly reduced dependence on manual interpretation by learning complex spatial and temporal patterns directly from seismic images and waveform data [5]. Similarly, deep neural network-based methods have been successfully applied to automatic channel detection and geological structure identification from large-scale seismic datasets [6]. These studies highlight the effectiveness of deep learning architectures in extracting meaningful representations from seismic data and improving interpretation accuracy across diverse geological environments.

Despite these advances, the existing literature remains fragmented across learning paradigms, application domains, data modalities, and evaluation strategies, making it difficult to establish consistent conclusions regarding model generalization, robustness, and operational reliability [7]. Furthermore, critical challenges—including data imbalance, limited transferability across geographical regions, uncertainty quantification, and model interpretability—are often investigated independently rather than within a unified analytical framework. This review synthesizes recent studies published between 2021 and 2025 on machine learning and deep learning methods for earthquake monitoring using both conventional seismic data and DAS measurements. The objective is to provide an integrated overview of recent methodological advances, practical limitations, and unresolved research challenges to support the development of robust, scalable, and intelligent seismic monitoring systems for next-generation earthquake hazard assessment [8,9].

The primary objective of this review is to systematically examine recent machine learning and deep learning approaches for earthquake monitoring, with particular emphasis on their application to Distributed Acoustic Sensing (DAS). Rather than presenting a broad overview of artificial intelligence in geoscience, this review specifically focuses on earthquake-related tasks, including seismic event detection, phase picking, earthquake characterization, magnitude estimation, and DAS-enabled seismic analysis. Furthermore, the review identifies current research gaps and discusses future directions for developing reliable, interpretable, and scalable intelligent seismic monitoring systems.

Although several review articles have summarized artificial intelligence applications in seismology, most have focused on specific topics such as seismic event detection, phase picking, earthquake early warning, or general machine learning applications in geophysics. In contrast, the present study provides a comprehensive and systematic synthesis of recent machine learning and deep learning developments for earthquake monitoring integrated with Distributed Acoustic Sensing (DAS), covering studies published between 2021 and 2025. Unlike previous reviews, this work unifies conventional seismic monitoring and DAS-based applications within a single analytical framework, categorizes recent advances according to learning paradigms and application domains, summarizes publicly available datasets, compares state-of-the-art methodologies, identifies current research challenges, and highlights future research directions, including uncertainty-aware learning, model generalization, interpretability, and real-time deployment. These contributions provide researchers and practitioners with an up-to-date reference for developing next-generation intelligent seismic monitoring systems. A comparison between existing review articles and the present systematic review is presented in Table 1, highlighting the scope, coverage, and key contributions of this study.

## 2. Fundamentals of Distributed Acoustic Sensing (DAS) for Earthquake Monitoring

### 2.1. DAS Working Principle

Distributed Acoustic Sensing (DAS) is an emerging fiber-optic sensing technology that transforms standard optical fiber cables into dense arrays of virtual seismic sensors. Unlike conventional seismometers that measure ground velocity or acceleration at discrete locations, DAS measures dynamic strain or strain-rate variations along the entire length of an optical fiber. The sensing mechanism is based on coherent laser pulses transmitted through the fiber, where a small portion of the light is continuously scattered back toward the interrogator unit. By analyzing temporal changes in the backscattered signal, DAS systems can detect ground vibrations and seismic wave propagation with meter-scale spatial resolution over distances extending to tens of kilometers. This capability enables continuous and high-density monitoring of seismic activity across large geographic regions using existing telecommunication or dedicated fiber-optic infrastructure.

### 2.2. Rayleigh Backscattering and Gauge Length

The operational principle of DAS primarily relies on Rayleigh backscattering, a naturally occurring optical phenomenon caused by microscopic refractive index variations within the fiber core. When laser pulses travel through the optical fiber, a fraction of the optical energy is scattered back toward the interrogator. Ground deformation induced by seismic waves changes the phase of the backscattered light, allowing the system to estimate strain variations along the fiber. A key DAS parameter is the gauge length, which defines the spatial interval over which strain measurements are averaged. Short gauge lengths provide higher spatial resolution but may increase measurement noise, whereas longer gauge lengths improve signal stability at the expense of spatial detail. The selection of an appropriate gauge length therefore represents an important trade-off in seismic monitoring applications.

### 2.3. DAS Architecture and Comparison with Seismometers

A typical DAS system consists of three main components: an optical interrogator unit, a fiber-optic sensing cable, and a data acquisition and processing platform. The interrogator generates coherent laser pulses and records the returned backscattered signals, while the fiber serves as a continuous sensing medium. Compared with traditional seismometer networks, DAS offers several advantages, including dense spatial sampling, large-area coverage, utilization of existing fiber infrastructure, and continuous real-time monitoring capabilities. However, DAS measurements represent strain rather than ground velocity, making direct comparison with conventional seismic recordings challenging. Additional limitations include large data volumes, sensitivity to environmental noise, directional sensing characteristics, and the need for specialized calibration procedures. These characteristics introduce unique challenges for machine learning models, particularly in earthquake detection, phase picking, localization, and magnitude estimation tasks.

Although many machine learning and deep learning algorithms have demonstrated remarkable performance on conventional seismic waveform data, their direct application to Distributed Acoustic Sensing (DAS) data remains challenging. Unlike conventional seismometers that record ground velocity or acceleration at discrete stations, DAS continuously measures distributed strain along optical fibers, producing high-dimensional spatial–temporal datasets with distinct signal characteristics, directional sensitivity, and significantly larger data volumes. Consequently, many models originally developed for conventional seismic networks require adaptation before they can be effectively applied to DAS data. Recent studies have addressed these challenges by incorporating spatial–temporal feature learning, self-supervised learning, transfer learning, Transformer-based architectures, and physics-informed approaches specifically designed for DAS measurements. Nevertheless, several limitations remain, including the scarcity of labeled DAS datasets, variability caused by fiber coupling conditions and installation environments, computational demands associated with large-scale DAS arrays, and limited model generalization across different geological settings. These challenges highlight the need for DAS-specific machine learning frameworks capable of exploiting the unique characteristics of distributed fiber-optic sensing while maintaining robust performance in real-world earthquake monitoring applications.

The increasing adoption of DAS in seismology has created new opportunities for artificial intelligence and machine learning applications. The massive volume, high spatial density, and complex noise characteristics of DAS measurements make traditional signal-processing techniques increasingly difficult to apply at scale. Consequently, machine learning and deep learning approaches have emerged as promising solutions for automated event detection, waveform classification, phase picking, source characterization, and magnitude estimation. The following sections review recent developments in these data-driven approaches and their applications in DAS-enabled seismic monitoring systems. Table 2 summarizes the key distinctions between conventional seismic monitoring systems and DAS-based sensing for earthquake magnitude estimation.

## 3. Challenges of Earthquake Magnitude Estimation Using DAS

Earthquake magnitude estimation using Distributed Acoustic Sensing (DAS) differs fundamentally from conventional magnitude estimation based on standard seismic networks. Traditional seismometers directly measure ground velocity or acceleration at discrete locations, and magnitude estimation procedures have been extensively developed and calibrated using these measurements over several decades. In contrast, DAS systems measure dynamic strain or strain rate along a continuous optical fiber, producing observations that differ both physically and geometrically from conventional seismic recordings.

One of the primary challenges arises from the fact that DAS measures strain rather than particle motion. Consequently, conventional magnitude-scaling relationships cannot be directly applied to DAS measurements without additional calibration and signal transformation procedures. Furthermore, the amplitude of DAS observations is strongly influenced by factors such as cable orientation, gauge length, ground coupling conditions, installation environment, and local geological properties. These factors can introduce significant variability in the recorded signals, even for seismic events of similar magnitude.

Another important distinction is the distributed nature of DAS measurements. Whereas conventional seismic stations provide observations at discrete sensor locations, DAS systems generate thousands of virtual sensing channels along a single fiber-optic cable. Although this dense spatial sampling offers unprecedented opportunities for high-resolution monitoring, it also introduces challenges related to data volume, redundancy, spatial correlation, and computational scalability. Consequently, machine learning models originally developed for conventional seismic networks often require adaptation before they can be effectively applied to DAS data.

Recent studies have demonstrated that machine learning and deep learning approaches can address some of these challenges by automatically learning complex relationships between DAS measurements and earthquake source characteristics. Convolutional neural networks, recurrent neural networks, Transformer-based models, and hybrid learning frameworks have shown considerable potential for extracting informative features from DAS waveforms and improving event characterization. Nevertheless, the development of reliable DAS-based magnitude estimation models remains an active area of research due to the limited availability of benchmark datasets, regional calibration requirements, and the need for uncertainty-aware prediction frameworks.

These differences demonstrate that earthquake magnitude estimation using DAS should not be considered a direct extension of conventional seismometer-based approaches. Instead, it represents a distinct research problem that requires specialized signal-processing techniques, calibration strategies, and machine learning models capable of handling the unique characteristics of distributed fiber-optic sensing data.

## 4. Literature Review

Supervised deep learning has remained the dominant paradigm for seismic event detection between 2021 and 2025, primarily because of the availability of labeled earthquake catalogs from regional and international seismic networks. Convolutional Neural Networks (CNNs) have been widely adopted for learning discriminative features from raw seismic waveforms and time–frequency representations, demonstrating improved detection accuracy and robustness compared with traditional signal-processing approaches [10]. Recent studies have further highlighted the effectiveness of deep learning techniques for seismic applications, including ground-motion prediction and seismic hazard assessment, where complex nonlinear relationships can be learned directly from large-scale datasets. Despite these advances, supervised models remain highly dependent on the quality, balance, and regional representativeness of labeled training data, which may limit their generalization to new geographic regions, varying noise environments, and emerging sensing technologies such as Distributed Acoustic Sensing (DAS) [11,12].

### 4.1. Deep Learning for Seismic Phase Picking and Arrival-Time Estimation

Accurate seismic phase picking remains a critical component of earthquake monitoring systems because it directly influences earthquake localization, magnitude estimation, and earthquake early warning performance. Recent advances in deep learning have significantly improved phase-picking accuracy by automatically learning complex waveform characteristics that are difficult to capture using traditional signal-processing techniques. Attention-based architectures have demonstrated the ability to simultaneously perform earthquake detection and P- and S-wave phase picking while effectively modeling long-range temporal dependencies in continuous seismic recordings [13]. Similarly, deep neural network-based approaches have achieved highly consistent arrival-time predictions across diverse tectonic settings, highlighting the robustness and generalization capability of data-driven phase-picking methods [14]. Further developments in generalized phase detection have improved model transferability across different seismic networks and monitoring environments, supporting broader applicability in operational seismic systems. Despite these advances, supervised phase-picking approaches remain dependent on the availability of high-quality labeled datasets and may experience performance degradation when applied to new geographic regions, varying noise conditions, or emerging sensing technologies such as Distributed Acoustic Sensing (DAS) [15].

### 4.2. Semi-Supervised and Unsupervised Learning Strategies

The increasing adoption of Distributed Acoustic Sensing (DAS) and large-scale seismic monitoring systems has generated vast volumes of seismic data, creating significant challenges for efficient data processing and interpretation. Consequently, recent research has increasingly explored machine learning approaches to improve the accuracy and efficiency of seismic monitoring tasks. Deep learning frameworks, such as RED-PAN, have demonstrated strong performance in seismic phase picking and event characterization, highlighting the potential of data-driven methods to improve the robustness and automation of seismic waveform analysis under diverse recording conditions [16]. Similarly, deep learning methods have been successfully utilized for structural damage assessment using seismic signals, highlighting their ability to learn informative representations from vibration and waveform data [17]. Machine learning has also contributed to understanding fault mechanics and forecasting failure processes by identifying hidden relationships between seismic observations and fault behavior. Furthermore, recent studies have employed machine learning techniques to analyze urban seismic noise, enabling improved characterization of environmental and anthropogenic noise sources in continuous monitoring systems. These developments demonstrate the growing role of machine learning in seismic and geophysical applications. However, challenges related to data quality, model generalization, and robust performance under varying noise conditions remain important areas for future research [18,19].

### 4.3. Hybrid Architectures for Spatiotemporal Seismic Analysis

Recent advances in deep learning have expanded the application of data-driven approaches across a wide range of environmental and geophysical monitoring tasks. Machine learning techniques have been increasingly employed in environmental seismology to improve the detection, classification, and interpretation of seismic signals generated by both natural and anthropogenic processes [20]. In volcanic monitoring, deep learning methods have demonstrated promising capabilities for classifying volcanic tremors and distinguishing between different types of volcanic activity, thereby supporting more effective hazard assessment and monitoring strategies [21]. Similarly, machine learning-based frameworks have been applied to volcano monitoring systems to enhance event detection, pattern recognition, and the interpretation of complex geophysical datasets [22]. Beyond earthquake and volcanic applications, data-driven seismic analysis has also been utilized for geothermal reservoir characterization, where machine learning models assist in identifying subsurface features and improving the understanding of reservoir dynamics from seismic observations [23]. Collectively, these studies demonstrate the expanding role of machine learning in geophysical monitoring and highlight its potential for extracting meaningful information from increasingly large and complex seismic datasets.

### 4.4. Attention-Based and Transformer Models

Recent studies have increasingly explored advanced machine learning techniques for continuous seismic monitoring and event characterization. Artificial intelligence has been successfully applied to volcano seismic monitoring, improving the detection, classification, and interpretation of volcanic seismic signals while supporting more efficient hazard assessment workflows [24]. Machine learning methods have also been employed to discriminate between induced and natural earthquakes, demonstrating their capability to identify subtle differences in seismic waveform characteristics that are often difficult to distinguish using conventional approaches [25]. Furthermore, deep learning frameworks have shown considerable promise for induced seismicity monitoring by enhancing the automated detection and characterization of seismic events in complex monitoring environments [26]. Similar advances have been reported in geothermal applications, where hybrid machine learning approaches have been utilized to improve the analysis and interpretation of geothermal-induced seismicity. Collectively, these studies highlight the growing importance of artificial intelligence in seismic monitoring and event characterization across a broad range of geophysical applications [27].

### 4.5. Bayesian Deep Learning and Uncertainty-Aware Models

Recent advances in machine learning have highlighted the importance of model reliability and robustness in operational seismic monitoring systems. Deep learning approaches have been increasingly applied to earthquake detection and characterization tasks, demonstrating improved performance in complex monitoring environments while supporting the automation of seismic analysis workflows [28]. In parallel, uncertainty quantification has emerged as an important research direction because reliable confidence estimates can reduce false alarms and improve decision-making in safety-critical applications. A comprehensive review of uncertainty-aware deep learning techniques has emphasized the potential of Bayesian methods, ensemble learning, and probabilistic modeling for estimating predictive uncertainty and improving model trustworthiness [29]. Furthermore, end-to-end deep learning frameworks have been proposed to streamline earthquake monitoring by jointly performing earthquake detection, seismic phase picking, and phase association within a unified architecture. By integrating these tasks into a single deep learning model, such approaches improve the efficiency, accuracy, and consistency of automated seismic event analysis while reducing the complexity associated with conventional multi-stage processing pipelines. These advances demonstrate the growing potential of deep learning to enhance real-time seismic monitoring and earthquake characterization [30].

### 4.6. Emerging Trends and Research Challenges

Recent research has increasingly focused on improving the generalization, robustness, and scalability of machine learning models for seismic applications. Hybrid deep learning architectures that combine convolutional and recurrent neural network components have demonstrated promising performance for seismic event classification by simultaneously capturing spatial waveform characteristics and temporal dependencies within seismic signals [31]. In parallel, advances in self-supervised and transfer learning have facilitated the development of deep learning frameworks for denoising DAS seismic data, reducing dependence on large labeled datasets while improving model adaptability across different monitoring environments [32]. Furthermore, comprehensive reviews of machine learning applications for seismic data interpretation have highlighted the growing adoption of data-driven approaches for subsurface geological feature identification while emphasizing persistent challenges related to dataset availability, model transferability, interpretability, and benchmarking standards [33]. Despite substantial progress, achieving robust cross-regional generalization, improving model transparency, and establishing standardized evaluation frameworks remain important research priorities for the broader adoption of machine learning in real-time seismic and DAS-based monitoring systems [34].

**Figure 1:**
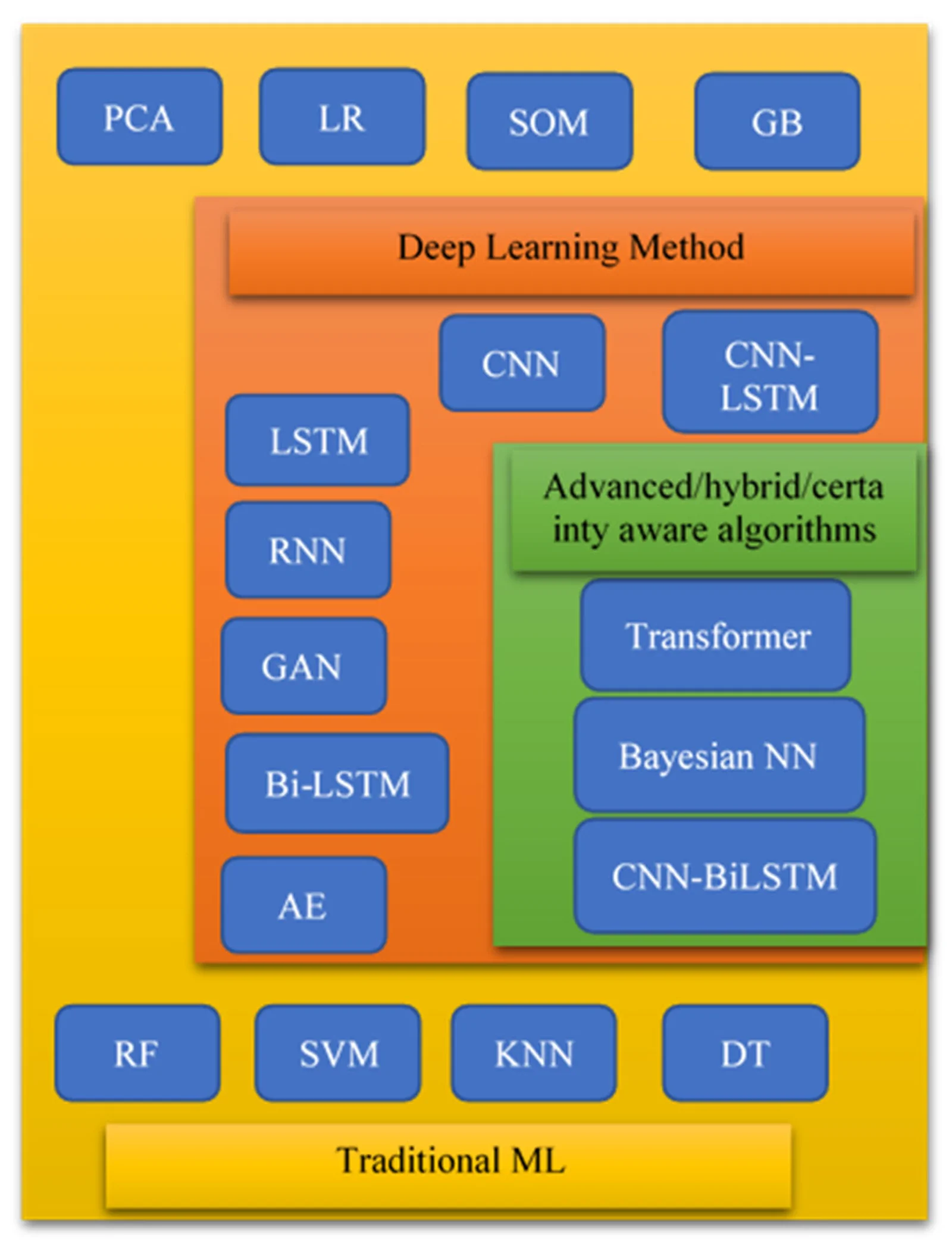
Taxonomy of machine learning algorithms employed in Distributed Acoustic Sensing (DAS)-based seismic monitoring. The figure summarizes the major categories of traditional machine learning, deep learning, hybrid, and uncertainty-aware approaches synthesized from the reviewed literature [31,36,37,38,39,40].

Figure 1 presents a hierarchical overview of the most widely adopted learning algorithms for seismic event detection using conventional seismic and DAS data. The algorithms are organized according to their level of maturity and adoption within the research community. Traditional supervised machine learning methods, including Random Forest (RF), Support Vector Machine (SVM), and K-Nearest Neighbor (KNN), were predominantly employed in earlier studies that relied on manually engineered time–frequency and statistical features. Although these methods are computationally efficient and relatively easy to interpret, their dependence on handcrafted features limits their effectiveness in noisy or complex environments and reduces their scalability. Subsequent research shifted toward deep learning architectures, particularly Convolutional Neural Networks (CNNs), Long Short-Term Memory (LSTM) networks, and Bidirectional LSTM (BiLSTM) models. These architectures learn directly from raw waveforms and spectrograms, enabling more accurate event detection by capturing both spatial and temporal patterns within seismic data. More recent studies have focused on hybrid and uncertainty-aware architectures, including Transformer networks, CNN–BiLSTM models with attention mechanisms, and Bayesian deep learning frameworks. These advanced models are capable of modeling long-range temporal dependencies, improving interpretability, and providing confidence estimates for predictions, thereby enhancing their reliability for real-world seismic monitoring applications [35].

**Figure 2:**
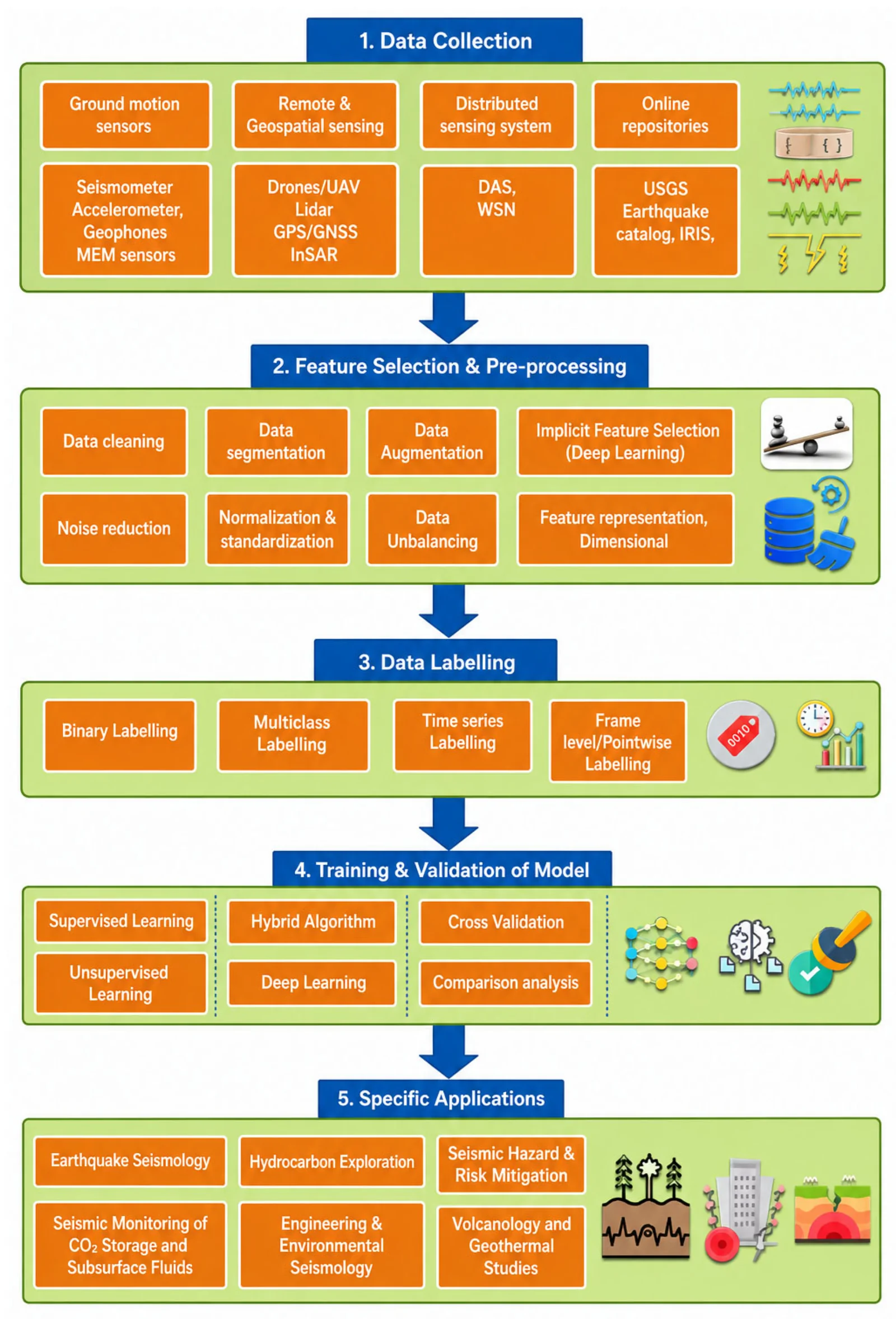
General workflow of machine learning and deep learning for seismic monitoring and earthquake engineering applications, illustrating the major stages of data collection, preprocessing, data labeling, model training and validation, and application domains. The figure was synthesized by the authors based on the reviewed literature [37,38,39,40].

Figure 2 illustrates a simplified workflow for seismic data analysis using machine learning, comprising five main stages: data acquisition, preprocessing, data labeling, model development, and application. Seismic data are acquired from a variety of sources, including seismometers, accelerometers, geophones, Micro-Electro-Mechanical Systems (MEMS), Unmanned Aerial Vehicles (UAVs), Light Detection and Ranging (LiDAR), Global Positioning System/Global Navigation Satellite System (GPS/GNSS), Interferometric Synthetic Aperture Radar (InSAR), Distributed Acoustic Sensing (DAS), and publicly available databases such as the United States Geological Survey (USGS) and the Incorporated Research Institutions for Seismology (IRIS). The preprocessing stage involves data cleaning, segmentation, denoising, normalization, and feature extraction to improve data quality and prepare the input for model training. The data labeling stage includes binary classification, multiclass classification, and time-series labeling, depending on the target application. Model development comprises the training and validation of machine learning and deep learning algorithms for seismic analysis. Finally, the developed models are applied to a wide range of applications, including earthquake monitoring, hydrocarbon exploration, seismic hazard assessment, carbon dioxide (CO_2_) storage monitoring, and environmental and geothermal studies.

## 5. Methodology

### 5.1. Search Strategy

This systematic review was conducted in accordance with the PRISMA 2020 guidelines (https://www.prisma-statement.org/prisma-2020, accessed on 1 July 2020) to ensure transparency, reproducibility, and methodological rigor in identifying and evaluating studies on the application of machine learning (ML), deep learning (DL), and Distributed Acoustic Sensing (DAS) for earthquake monitoring, seismic event detection, and related seismic analysis tasks. A comprehensive literature search was performed across four major academic databases: IEEE Xplore, Web of Science (WoS), Scopus, and Google Scholar. The search included peer-reviewed journal articles and conference papers published between January 2021 and December 2025. To maximize retrieval accuracy, the search strategy employed Boolean operators (AND, OR), database-specific search syntax, field restrictions (title, abstract, and keywords), and publication filters.

The search strategy was iteratively refined through multiple pilot searches to achieve an appropriate balance between search sensitivity and specificity. Boolean operators (AND, OR) and database-specific search syntaxes were employed to ensure comprehensive retrieval of relevant studies while minimizing the inclusion of irrelevant records. Search terms were organized into three thematic domains. The seismology domain included the keywords “seismic data,” “seismic wave,” “microseismic,” “earthquake event,” “earthquake monitoring,” and “earthquake prediction.” The Distributed Acoustic Sensing (DAS) domain comprised “distributed acoustic sensing,” “fiber-optic cable,” “optical fiber sensing,” “DAS data,” and “DAS technology.” The machine learning domain included “machine learning,” “deep learning,” “artificial intelligence,” “neural network,” “convolutional neural network (CNN),” “LSTM,” “BiLSTM,” “Transformer,” and “Bayesian learning.” These keywords were systematically combined using Boolean operators to maximize search coverage across the selected databases. Appendix B contains the keyword groups used in the search strategy.

No geographical restrictions were applied during the literature search to ensure comprehensive representation of global research. Consequently, the final dataset included studies from major research regions, including North America, Europe, and Asia, providing a comprehensive overview of recent advances in machine learning, deep learning, and Distributed Acoustic Sensing for earthquake monitoring. The complete Boolean search strings used for each database are presented in Table 3.

The search strategy was adapted to the syntax requirements of each database while maintaining a consistent conceptual framework. Boolean operators (AND, OR), phrase searching using quotation marks, and database-specific search fields were employed to maximize retrieval accuracy. Searches were conducted within the title, abstract, and keyword fields whenever supported by the respective database. Filters were applied to include only English-language, peer-reviewed journal articles and conference papers published between 2021 and 2025. The literature search was conducted between December 2025 and January 2026, with the final database search completed in January 2026.

### 5.2. Study Process Style (PRISMA)

The study selection process followed the PRISMA 2020 framework and consisted of five sequential stages.

#### 5.2.1. Identification

A total of 252,223 records were initially retrieved from IEEE Xplore, Web of Science, Scopus, and Google Scholar using the predefined Boolean search strategy. This number represents the complete search output generated by the selected databases prior to the application of any screening or eligibility criteria and does not correspond to the number of studies manually reviewed. To obtain a relevant and manageable dataset, publication year (2021–2025), document type (peer-reviewed journal articles and conference papers), English language, and relevance filters were first applied within each database. Duplicate records were subsequently identified and removed, followed by title and abstract screening to exclude studies unrelated to earthquake monitoring, Distributed Acoustic Sensing (DAS), or machine learning and deep learning applications. The remaining articles were then subjected to full-text eligibility assessment and quality evaluation according to the predefined inclusion and exclusion criteria, resulting in the final selection of 138 studies included in this systematic review.

#### 5.2.2. Screening

Following the application of publication and relevance filters, 63,717 records remained for title and abstract screening. During this stage, studies unrelated to earthquake monitoring, DAS, or machine learning applications were excluded. After duplicate removal and relevance screening, 938 studies were retained for full-text eligibility assessment.

#### 5.2.3. Eligibility

A total of 938 full-text articles were assessed for eligibility based on methodological rigor, relevance to machine learning and DAS applications, data availability, and the completeness of experimental evaluation.

#### 5.2.4. Exclusion

Of the 938 full-text articles assessed for eligibility, 800 studies were excluded because they did not satisfy the predefined inclusion criteria. The primary reasons for exclusion included the absence of empirical validation, insufficient methodological detail, lack of relevance to earthquake monitoring or Distributed Acoustic Sensing (DAS), and inadequate reporting of experimental results.

#### 5.2.5. Inclusion

Following the eligibility assessment and quality evaluation, 138 studies satisfied the predefined inclusion criteria and were included in the final qualitative synthesis and comprehensive analysis.

### 5.3. Data Extraction and Synthesis

For each eligible study, relevant information was systematically extracted using a standardized data extraction framework. The extracted information included:

(1) Publication details, including publication year, authors, and publication source.
(2) The type of data used, such as conventional seismic data, Distributed Acoustic Sensing (DAS) data, or hybrid datasets.
(3) The machine learning or deep learning techniques employed, including Convolutional Neural Networks (CNNs), Long Short-Term Memory (LSTM) networks, Bidirectional LSTM (BiLSTM), Transformer models, Bayesian learning methods, and other artificial intelligence approaches.
(4) The primary application of the proposed method, including seismic event detection, earthquake magnitude estimation, waveform classification, phase picking, earthquake early warning, and related seismic monitoring tasks.
(5) Performance evaluation metrics reported in each study, including accuracy, precision, recall, F1-score, Root Mean Square Error (RMSE), and uncertainty estimation, where applicable.

The extracted studies were subsequently synthesized and categorized into three major research themes:

(1) Machine Learning and DAS for Seismic Event Detection
(2) Seismic Waveform Classification and Feature Extraction
(3) Hybrid Deep Learning Models for Earthquake Prediction

### 5.4. Assessment of Quality

To ensure methodological rigor, a quality assessment framework was developed based on four evaluation criteria: reproducibility, model transparency, validation strategy, and performance reporting. Reproducibility was assessed based on the availability of datasets, source code, or sufficiently detailed methodological descriptions. Model transparency was evaluated based on whether studies incorporated explainability techniques or uncertainty quantification methods. Validation strategy assessed the use of appropriate evaluation procedures, such as cross-validation, independent test datasets, or Bayesian validation approaches. Performance reporting examined the completeness, consistency, and clarity of the reported evaluation metrics.

The quality assessment framework employed a binary scoring scheme (1 = criterion satisfied; 0 = criterion not satisfied), with a maximum possible quality score of 4. The normalized quality score was calculated as:

$$Quality\;Score=\frac{Total\;score}{4}\times 100$$

Studies achieving a quality score of 70% or higher (i.e., satisfying at least three of the four evaluation criteria) were considered to have sufficient methodological quality for inclusion in the final qualitative synthesis. Studies that did not meet this threshold were excluded because of insufficient methodological rigor or inadequate reporting quality. Table 4 summarizes the quality assessment framework and scoring criteria.

Each study was assessed against the predefined quality assessment criteria during the screening and eligibility evaluation process. A quality threshold of ≥70%, corresponding to satisfying at least three of the four evaluation criteria, was adopted to ensure methodological rigor. Only studies meeting this predefined quality threshold were retained for the final qualitative synthesis, whereas studies that did not satisfy the required methodological quality standards were excluded. Consequently, the final review included 138 peer-reviewed studies that met both the eligibility and quality assessment requirements.

### 5.5. Inclusion and Exclusion Criteria

The study selection process was guided by predefined inclusion and exclusion criteria to ensure the relevance, quality, and consistency of the reviewed literature. These criteria were developed to identify studies specifically investigating machine learning and deep learning applications for earthquake monitoring, seismic data analysis, and Distributed Acoustic Sensing (DAS). In addition to publication year, publication type, language, and full-text availability, each study was evaluated based on its methodological transparency, dataset description, experimental validation, and reporting of performance metrics. Only studies providing sufficient methodological and technical detail to enable meaningful comparison, critical evaluation, and systematic synthesis were included in the final review. The complete inclusion and exclusion criteria applied during the screening process are presented in Table 5.

**Figure 3:**
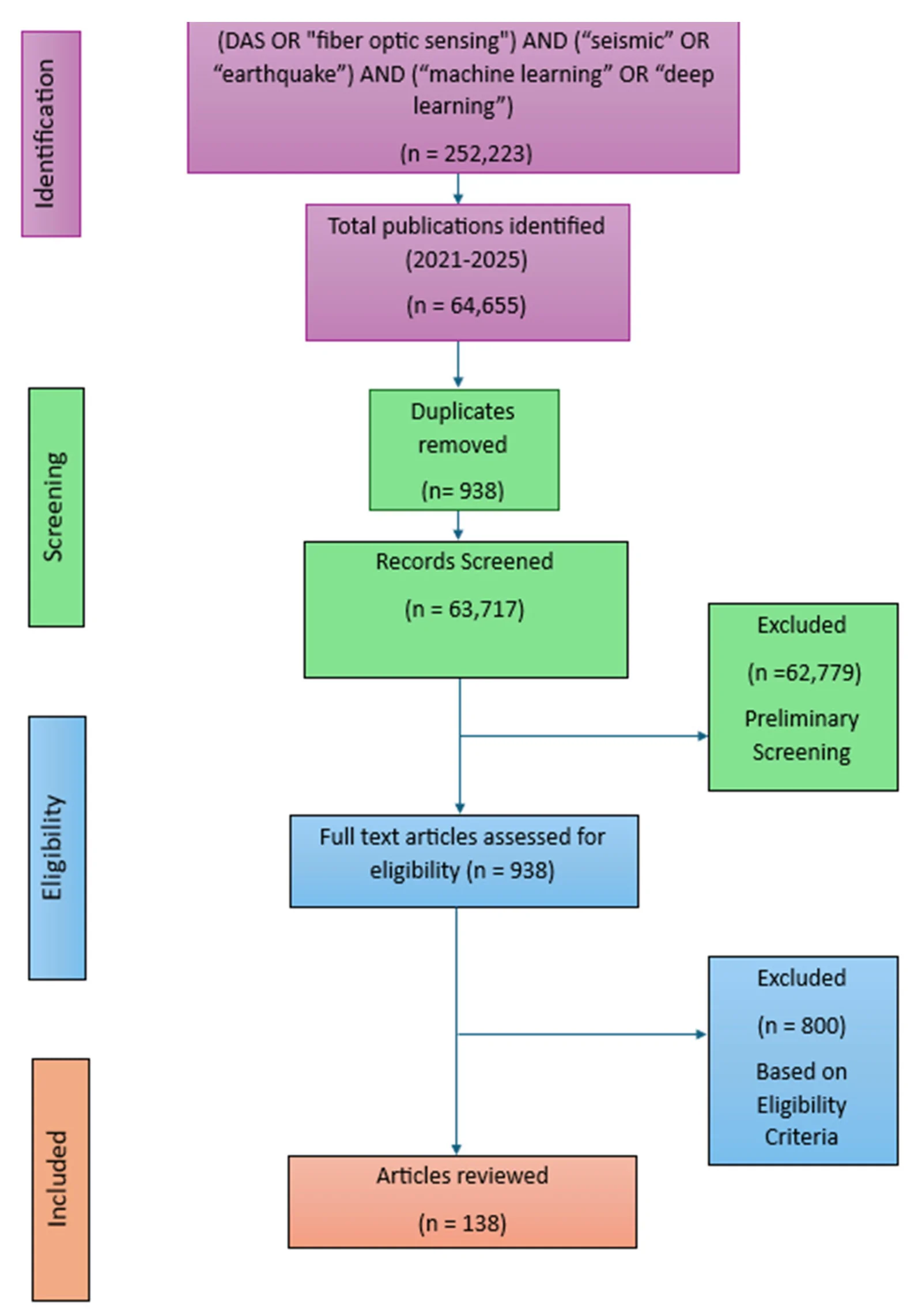
PRISMA flow diagram of the literature search and selection process.

The overall screening and selection process are illustrated in Figure 3.

A summary of the literature search and screening process across the selected databases is presented in Table 6. The table highlights the progressive refinement of records from initial retrieval to final inclusion, ensuring transparency and consistency in the study selection process.

The final set of 138 studies comprised both core and supporting publications that contributed to the comprehensive analysis of machine learning and deep learning applications in earthquake monitoring and Distributed Acoustic Sensing (DAS)-based seismology. Table 7 provides a summary of the studies included in the systematic literature review conducted in this research.

The search queries were iteratively refined to improve the relevance of the retrieved studies while minimizing the inclusion of unrelated publications. Duplicate records identified across the selected databases were removed using [Zotero/Mendeley] reference management software. Subsequently, the titles and abstracts of the retrieved studies were independently screened according to the predefined inclusion and exclusion criteria in accordance with the PRISMA 2020 workflow. Studies that satisfied the initial screening criteria were subsequently subjected to full-text assessment for eligibility.

## 6. Results

### 6.1. Keyword Distribution

**Figure 4:**
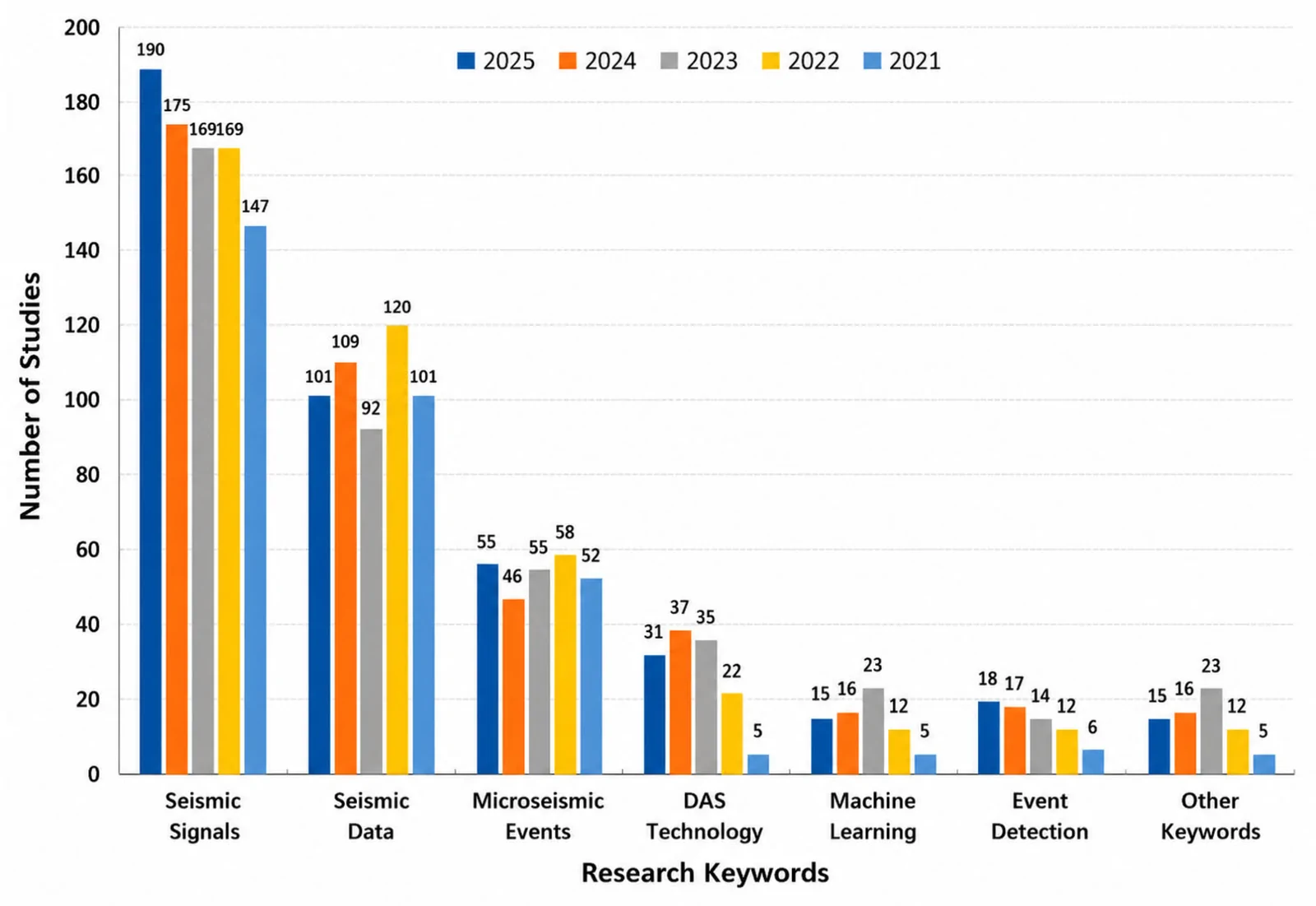
Annual distribution of raw keyword-based search results retrieved from the literature search (2021–2025) across the major research keywords. The values represent pre-screening search records identified before duplicate removal and eligibility assessment and do not correspond to the final cohort of 138 studies included in this systematic review.

Figure 4 presents the annual distribution of raw keyword-based search results retrieved during the literature search between 2021 and 2025. These publication counts represent the initial search records obtained before duplicate removal, title and abstract screening, and full-text eligibility assessment, and therefore should not be interpreted as the final set of studies included in this systematic review. Following the PRISMA screening process, 138 studies satisfied the inclusion criteria and were subsequently analyzed.

The keyword search results indicate a steady increase in publications related to seismic signals, seismic data, microseismic events, Distributed Acoustic Sensing (DAS), machine learning, and event detection throughout the study period. Most keyword categories reached their highest search volumes in 2025, reflecting the growing research interest in artificial intelligence-driven approaches for earthquake monitoring and seismic analysis.

Among the retrieved records, seismic signals and seismic data produced the largest number of search results, followed by Distributed Acoustic Sensing (DAS), machine learning, and event detection. This trend highlights the increasing research activity in data-driven seismic monitoring and geophysical applications. Although microseismic events generated comparatively fewer search results, they exhibited a consistent upward trend, demonstrating the expanding application of DAS in geothermal reservoir monitoring, mining, carbon dioxide (CO_2_) storage monitoring, and structural health monitoring.

Overall, the keyword search trends illustrate the rapid growth of research related to machine learning and Distributed Acoustic Sensing, particularly since 2023. These search statistics were used to characterize the evolution of research topics, whereas the detailed qualitative and quantitative analyses presented in the remainder of this review are based exclusively on the 138 studies that met the predefined inclusion criteria.

### 6.2. Classification of Reviewed Studies Based on Application Areas in Seismic Monitoring

Table 8 presents the classification of the reviewed studies according to their primary application areas in seismic monitoring and analysis. The studies are categorized into key domains, including earthquake seismology, hydrocarbon exploration, seismic hazard assessment, carbon dioxide (CO_2_) storage monitoring, and environmental and engineering seismology. This classification highlights the broad applicability of machine learning and deep learning techniques across diverse seismic applications, demonstrating their contributions to both conventional seismological research and emerging fields such as induced seismicity and subsurface monitoring. Overall, the table provides a structured overview of the application of ML and DL approaches across various seismic problem domains, illustrating the expanding role of artificial intelligence in modern seismic monitoring and analysis.

### 6.3. Articles Published by Journal

**Figure 5:**
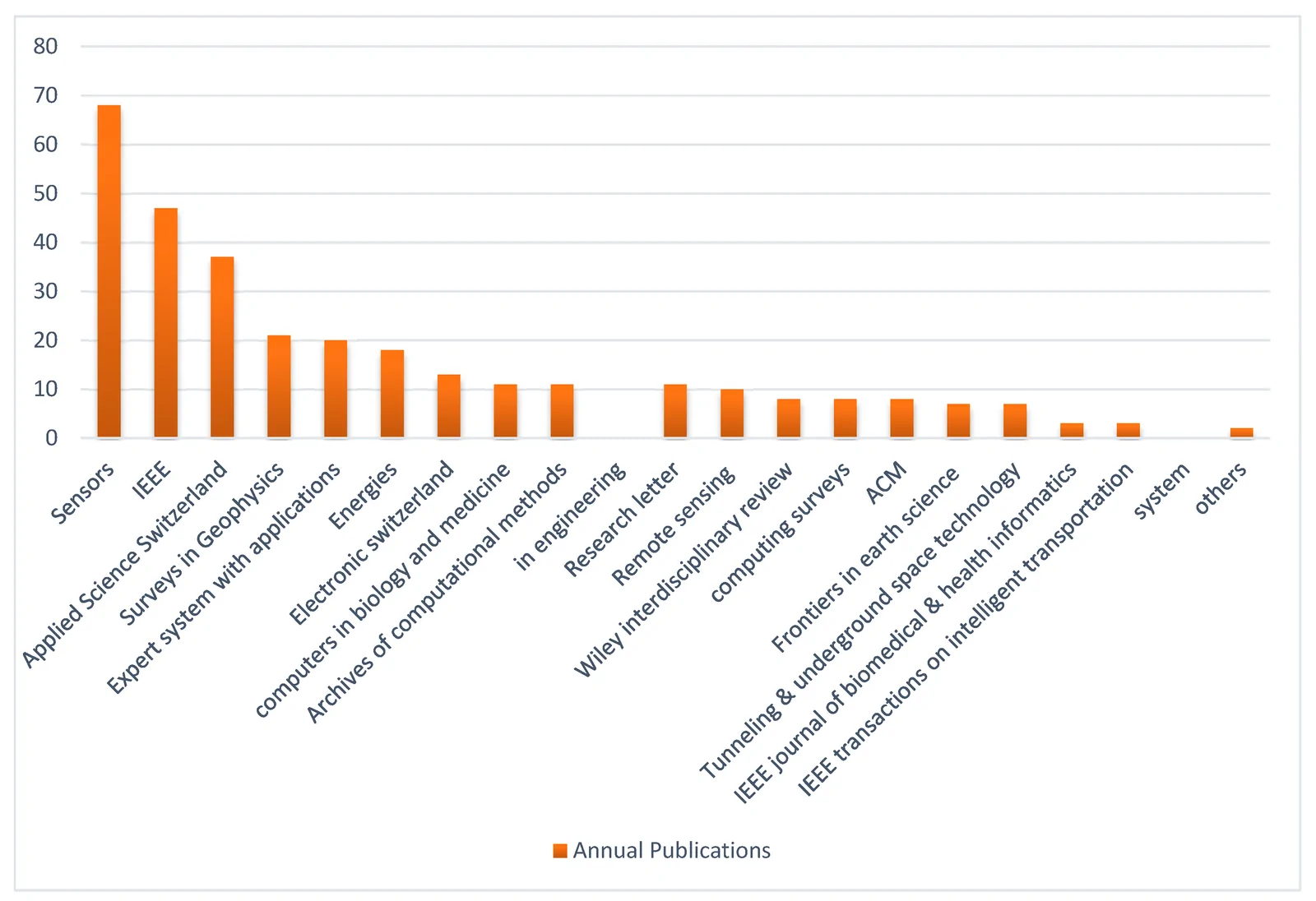
Distribution of reviewed publications across different journals and sources, highlighting that Sensors and IEEE journals contribute the largest share of articles in this research area.

Figure 5 illustrates the distribution of publications across major journals and conference proceedings between 2021 and 2025. The publication pattern indicates that research activity is concentrated within a relatively small number of high-impact journals and conferences, reflecting both the maturity and interdisciplinary nature of seismic sensing and intelligent signal-processing research. Sensors emerged as the most prolific publication venue, highlighting the growing importance of sensor-based technologies, data-driven methodologies, Distributed Acoustic Sensing (DAS), Internet of Things (IoT)-enabled monitoring, and high-resolution seismic measurements. IEEE publications, including IEEE Access and various IEEE Transactions journals, also exhibited substantial publication activity, demonstrating the increasing interest of the signal processing, machine learning, and intelligent systems communities in seismic monitoring applications.

Mid-tier journals, such as Applied Sciences, Surveys in Geophysics, and Expert Systems with Applications, consistently contributed research on computational frameworks, machine learning models, and application-oriented seismic analysis. In contrast, the relatively smaller number of publications in specialized journals indicates that, although research efforts continue across diverse publication venues, a substantial proportion of influential contributions remains concentrated within technically focused journals. Overall, these publication trends suggest that seismic sensing research is expanding rapidly while remaining centered on journals that integrate sensing technologies, artificial intelligence, and machine learning for advanced seismic monitoring.

### 6.4. Articles Published by Country-Wise

**Figure 6:**
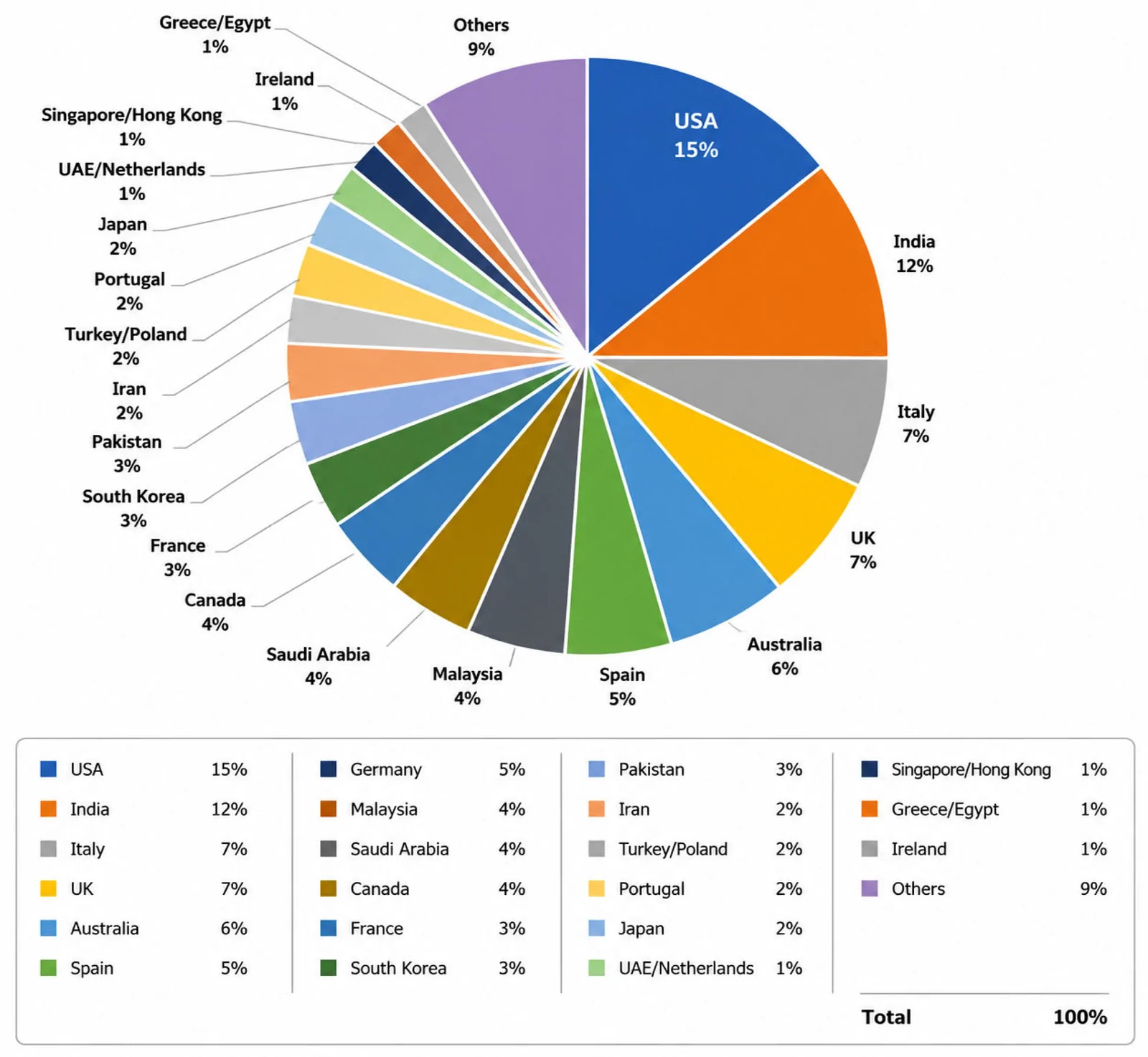
Country-wise distribution of publications retrieved from the selected international English-language databases (2021–2025).

Figure 6 presents the country-wise distribution of publications retrieved from the selected international English-language databases, illustrating the geographical distribution of research on machine learning (ML), deep learning (DL), and Distributed Acoustic Sensing (DAS) for seismic monitoring. The United States accounts for the largest share of publications, reflecting its long-standing investment in seismic monitoring infrastructure, computational geophysics, and data-driven approaches to earthquake hazard assessment and mitigation. Significant research contributions are also observed from India and Italy, where increasing emphasis has been placed on integrating artificial intelligence with dense seismic monitoring networks.

European countries, including the United Kingdom, Germany, France, Italy, Spain, and Portugal, exhibit consistent research activity supported by well-established geophysical research infrastructures and internationally recognized research institutions. In addition, countries such as Australia, Malaysia, Saudi Arabia, Canada, Iran, Pakistan, South Korea, Japan, and Turkey also contribute to the growing body of research, demonstrating the widespread adoption of machine learning, deep learning, and Distributed Acoustic Sensing (DAS) technologies for seismic monitoring and related geophysical applications. Collectively, these findings reflect the increasing international research interest represented in the selected English-language databases and highlight the expanding application of artificial intelligence-driven approaches for earthquake monitoring and seismic analysis. Overall, the geographical distribution of publications reflects the international research activity represented in the selected English-language databases. Because publication trends are influenced by database coverage, language restrictions, research funding, data availability, seismic activity, and regional research priorities, these findings should be interpreted as an overview of research represented in the selected databases rather than a comprehensive measure of worldwide research activity or country-level research leadership.

### 6.5. Method Category Distribution

**Figure 7:**
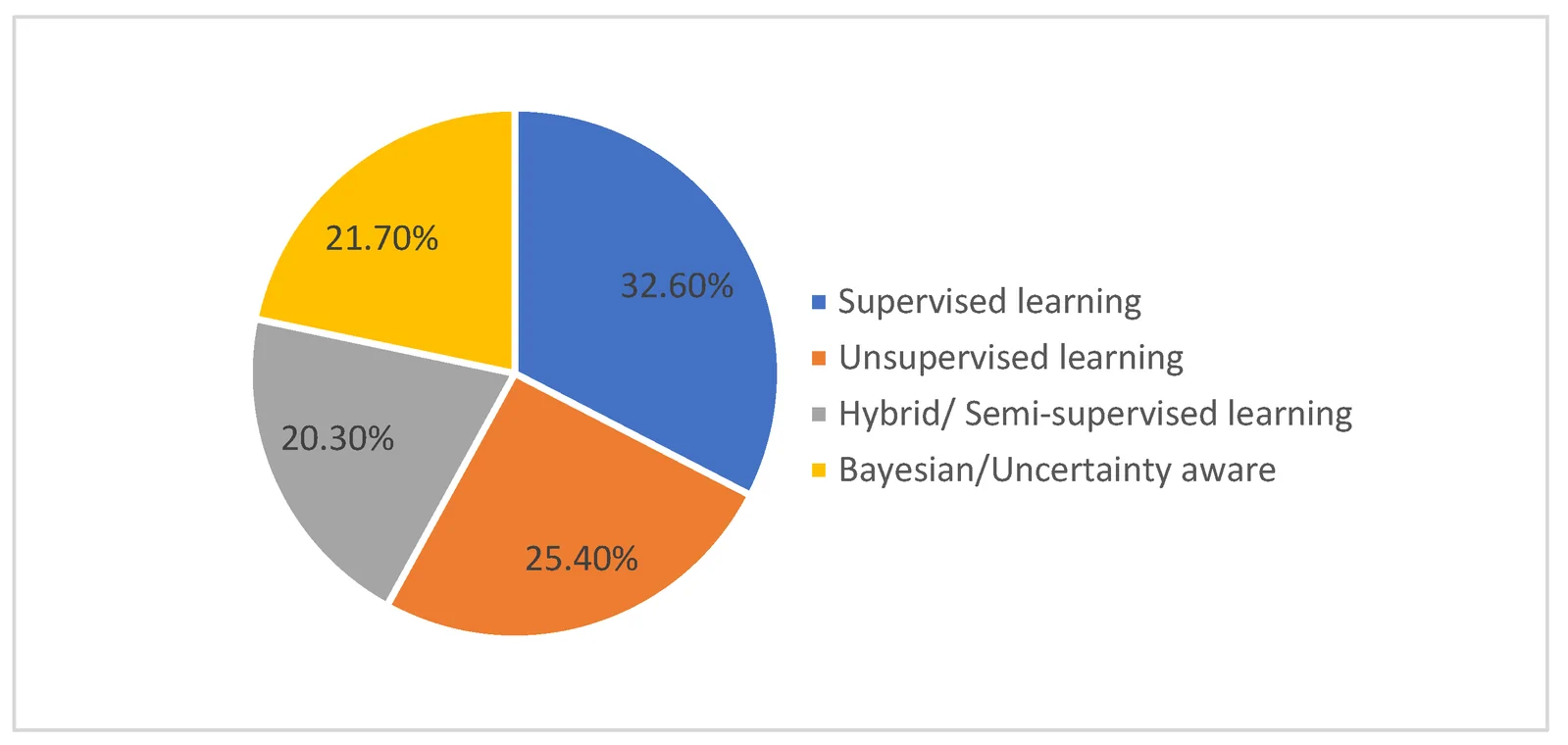
Distribution of machine learning paradigms across the 138 studies included in this systematic review. Supervised learning represents the largest category (32.6%), followed by unsupervised learning (25.4%), Bayesian/uncertainty-aware learning (21.7%), and hybrid/semi-supervised learning (20.3%). The percentages are derived directly from the final study cohort summarized in Table 9.

Figure 7 presents the distribution of machine learning paradigms adopted in the 138 studies included in this systematic review. Supervised learning represents the largest category, accounting for 32.6% (45 studies) of the reviewed literature. This predominance reflects the widespread availability of labeled benchmark datasets and the continued reliance on supervised models for seismic event detection, phase picking, earthquake classification, magnitude estimation, and related monitoring tasks.

Unsupervised learning constitutes 25.4% (35 studies) of the reviewed papers. These approaches are increasingly employed for anomaly detection, feature representation, clustering, and pattern discovery in large-scale unlabeled Distributed Acoustic Sensing (DAS) datasets, thereby reducing the dependence on manually annotated seismic records.

Bayesian and uncertainty-aware learning accounts for 21.7% (30 studies), highlighting the growing emphasis on prediction confidence and uncertainty quantification in safety-critical seismic applications. Techniques such as Monte Carlo Dropout, Bayesian Neural Networks, and Deep Ensembles are increasingly being investigated to improve model reliability and support trustworthy decision-making in earthquake early-warning and real-time seismic monitoring systems.

Hybrid and semi-supervised learning represent 20.3% (28 studies) of the reviewed literature. These methods combine complementary deep learning architectures or integrate supervised and unsupervised learning strategies to improve feature extraction, temporal modeling, robustness, and predictive performance, particularly when labeled seismic data are limited.

Overall, the distribution presented in Figure 7 demonstrates that the reviewed literature encompasses a diverse range of learning paradigms. While supervised learning remains the most widely adopted approach, there is a clear and increasing research interest in Bayesian uncertainty-aware methods and hybrid learning frameworks to address the challenges of complex seismic signals, limited labeled data, and the need for reliable operational monitoring in Distributed Acoustic Sensing applications.

**Table 9:** Types of ML Algorithms.

ML Type	Algorithm	Publication Count
Supervised Learning	CNN, LSTM/BiLSTM, GRU, classification, regression, transformer	45
Unsupervised Learning	VAE, Autoencoder, Isolation Forest, clustering	35
Hybrid/Semi Supervised Learning	Unsupervised pretraining+supervised fine-tuning, ensemble mixing	28
Bayesian/Uncertainty-aware Model	MC dropout, Bayesian Neural Networks, Deep Ensembles	30

### 6.6. Taxonomy of Machine Learning Approaches Across the Reviewed Studies

Table 9 presents a taxonomy of the machine learning approaches adopted in the reviewed studies. The results indicate that hybrid learning approaches are among the most widely employed methodologies, reflecting the increasing emphasis on improving model robustness, predictive accuracy, and generalization in complex seismic environments. These approaches typically combine complementary learning techniques to enhance feature extraction, representation learning, and predictive performance under challenging conditions, including noisy seismic data, limited labeled datasets, and class imbalance. This trend highlights the growing transition toward flexible, data-driven, and performance-oriented artificial intelligence frameworks for earthquake monitoring and seismic analysis, demonstrating the increasing adoption of hybrid learning approaches in modern intelligent seismic monitoring systems.

### 6.7. Annual Publication Trend of Method Evaluation

**Figure 8:**
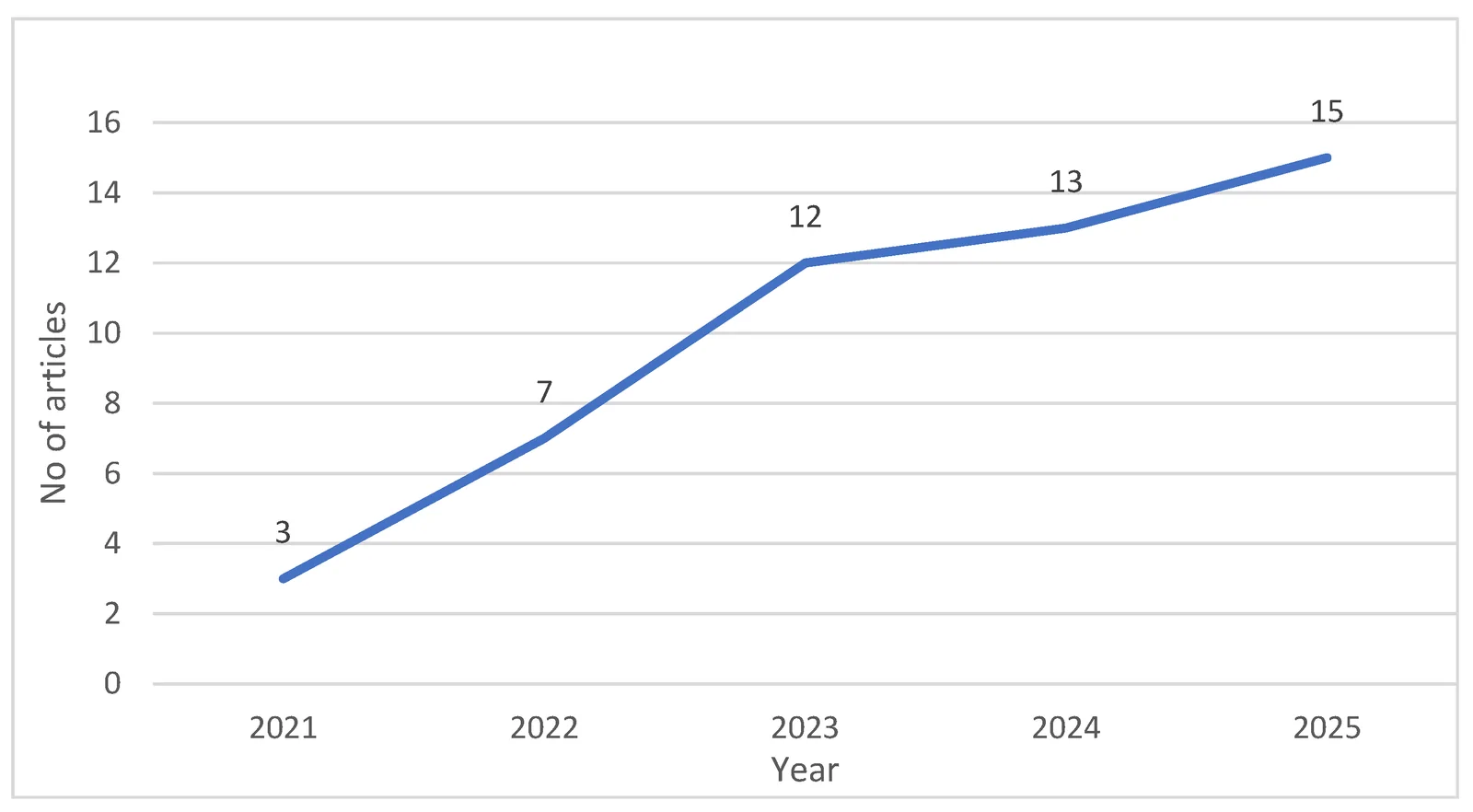
Annual publication trend from 2021 to 2025, indicating growing interest in machine learning and deep learning for seismic data analysis.

Figure 8 illustrates the annual publication trend between 2021 and 2025, demonstrating a clear and consistent increase in the number of published studies over the review period. The number of publications increased from approximately three studies in 2021 to around fifteen studies in 2025, indicating sustained growth in research activity. The most pronounced increase occurred between 2022 and 2023, followed by a steady upward trend through 2024 and 2025. This publication pattern reflects the growing academic interest in machine learning, deep learning, and Distributed Acoustic Sensing (DAS) for earthquake monitoring. The observed trend suggests that what initially emerged as a relatively specialized research area has evolved into an increasingly active field, attracting broader attention and continued investigation from the international research community.

### 6.8. Distribution of Hybrid and Deep Learning Models in Seismic Applications

**Figure 9:**
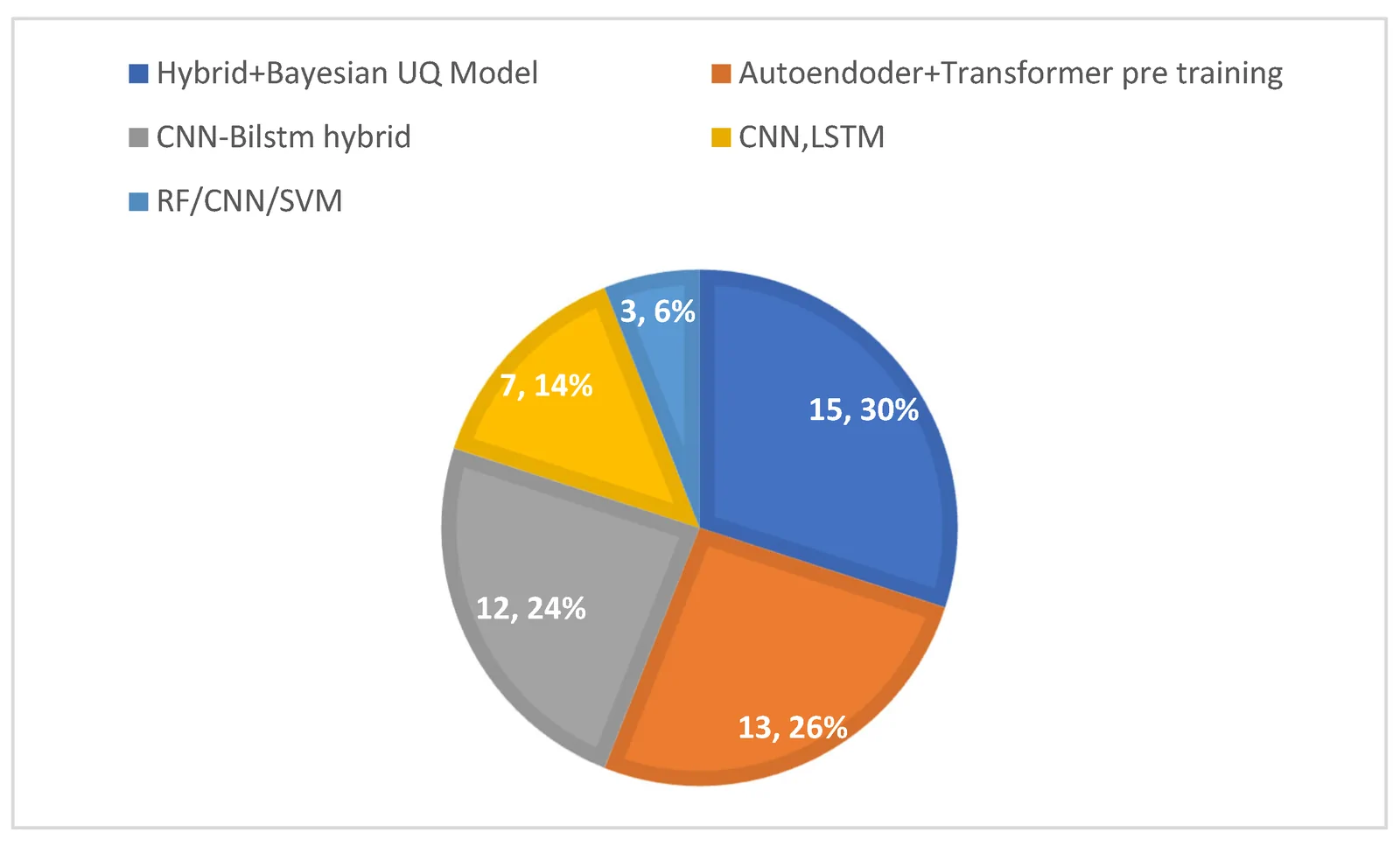
Distribution of model architectures, highlighting the dominance of hybrid and uncertainty-aware approaches.

Figure 9 presents the distribution of machine learning and deep learning model architectures employed in the reviewed studies. Hybrid models, particularly those incorporating uncertainty quantification, represent the largest proportion of the reviewed literature, followed by Transformer-based and ensemble learning approaches. In contrast, classical machine learning algorithms and standalone deep learning models account for comparatively smaller proportions. This distribution reflects the growing preference for hybrid, uncertainty-aware, and robust modeling strategies capable of addressing the complexity, variability, and uncertainty associated with seismic data. Overall, the results indicate a clear shift toward integrating multiple learning paradigms to enhance model accuracy, generalization, and reliability in modern seismic monitoring applications.

### 6.9. Comparative Evaluation of ML and DL Models for DAS-Based Seismic Monitoring

A structured comparative analysis of the machine learning and deep learning models employed in seismic monitoring is presented in Table 10. The table summarizes the key approaches by highlighting their strengths, limitations, research gaps, and reported performance metrics, including accuracy, Root Mean Square Error (RMSE), and coefficient of determination (R^2^). This comparative analysis provides a systematic basis for evaluating the effectiveness, robustness, and applicability of these models in Distributed Acoustic Sensing (DAS)-based seismic monitoring. Furthermore, it facilitates the identification of current research challenges and supports the selection of appropriate machine learning techniques for future DAS applications.

The comparative analysis presented in Table 10 indicates that deep learning models, particularly Convolutional Neural Networks (CNNs), Long Short-Term Memory (LSTM) networks, and Transformer-based architectures, consistently achieve superior performance in seismic monitoring tasks because of their ability to capture complex spatial and temporal patterns in seismic data. Hybrid models, such as CNN–LSTM and Transformer-based frameworks, further enhance predictive performance by combining efficient feature extraction with sequential pattern learning. In particular, hybrid models incorporating Bayesian uncertainty estimation demonstrate strong predictive capability while simultaneously providing confidence measures, which are essential for real-time seismic decision-making and earthquake early warning systems. Despite their superior performance, these models exhibit several limitations, including high computational complexity, dependence on large labeled datasets, and limited interpretability. Conventional machine learning approaches, such as Support Vector Machines (SVMs) and Random Forests, remain valuable because of their computational efficiency and interpretability; however, they are generally less effective in handling large-scale and complex seismic datasets. Furthermore, the comparative analysis highlights several important research gaps, including the lack of standardized benchmark datasets, limited cross-regional validation, and insufficient integration of uncertainty quantification. Addressing these challenges through hybrid architectures, self-supervised learning, and physics-informed models represents a promising direction for future research in Distributed Acoustic Sensing (DAS)-based seismic monitoring.

The primary contribution of this review lies in integrating recent advances in machine learning, deep learning, and Distributed Acoustic Sensing (DAS) within a unified systematic framework. By critically comparing existing methodologies, datasets, applications, and emerging research trends, this review identifies unresolved challenges and provides practical guidance for future research on artificial intelligence-enabled seismic monitoring systems.

## 7. Future Research Directions

Despite Although significant progress has been achieved in applying machine learning and deep learning to Distributed Acoustic Sensing (DAS)-based seismic monitoring, several important research challenges remain. One of the primary limitations is the absence of standardized benchmark datasets, evaluation protocols, and performance metrics, making objective comparisons among different algorithms difficult. Future studies should prioritize the development of unified benchmarking frameworks that employ consistent evaluation metrics, including Accuracy, Precision, Recall, F1-score, Root Mean Square Error (RMSE), and the coefficient of determination (R^2^), to facilitate fair, reproducible, and transparent model comparisons.

Another important direction is the transition from offline retrospective analysis to real-time operational deployment. Future machine learning frameworks should be capable of processing continuous DAS data streams while maintaining high computational efficiency, robustness, and low-latency inference for earthquake early warning and continuous seismic monitoring applications. Furthermore, models should be designed to generalize across different fiber-optic cable types, installation environments, coupling conditions, and geological settings to improve practical applicability.

Multimodal data integration represents another promising research direction. Combining DAS measurements with complementary sensing technologies, including Global Navigation Satellite System (GNSS) observations, conventional seismometers, accelerometers, Internet of Things (IoT) sensors, satellite observations, and other geophysical monitoring systems, has the potential to improve event detection, source localization, earthquake magnitude estimation, and overall monitoring reliability.

Future research should also emphasize the development of interpretable, explainable, and uncertainty-aware artificial intelligence models. Although recent deep learning approaches have achieved remarkable predictive performance, many existing methods provide deterministic predictions without quantifying confidence. Bayesian deep learning techniques, Monte Carlo Dropout, Bayesian Neural Networks, and other uncertainty quantification approaches offer promising solutions for improving prediction reliability, particularly in noisy environments and safety-critical earthquake monitoring applications.

In addition, increasing attention should be devoted to hybrid and foundation models capable of efficiently learning from large-scale seismic datasets. The integration of convolutional neural networks, recurrent neural networks, Transformers, graph neural networks, self-supervised learning, and large foundation models may further improve feature representation, temporal modeling, scalability, and transferability across different seismic monitoring tasks.

Overall, future research should focus on developing robust, scalable, interpretable, uncertainty-aware, and reproducible machine learning frameworks supported by standardized datasets and evaluation protocols. Addressing these challenges will facilitate the deployment of trustworthy artificial intelligence systems for next-generation DAS-based seismic monitoring, earthquake early warning, hazard assessment, and other geophysical applications.

## 8. Conclusions

This systematic review critically synthesizes recent advances in machine learning and deep learning for seismic analysis, with particular emphasis on earthquake monitoring, Distributed Acoustic Sensing (DAS), and related geophysical applications published between 2021 and 2025. The reviewed literature demonstrates a significant paradigm shift from conventional rule-based and signal-processing techniques toward data-driven deep learning approaches capable of learning complex spatial and temporal representations directly from seismic waveforms. Deep learning architectures, particularly CNN–RNN hybrid models, have demonstrated remarkable performance in seismic event detection, phase picking, waveform classification, and earthquake characterization, achieving higher accuracy and robustness under noisy and heterogeneous recording conditions. More recently, attention-based and Transformer architectures have further improved the modeling of long-range temporal dependencies while providing greater scalability for continuous, high-dimensional seismic data streams.

Despite these advances, this review identifies several persistent challenges that continue to limit the practical deployment of machine learning models in real-world seismic monitoring systems. These challenges include a strong dependence on labeled datasets, limited model generalization across geographical regions and sensing technologies, insufficient model interpretability, and inadequate uncertainty quantification for safety-critical applications. The increasing adoption of Distributed Acoustic Sensing (DAS) further amplifies these challenges because of the massive volume of generated data, complex noise characteristics, and limited availability of annotated datasets. Consequently, there is growing interest in self-supervised, semi-supervised, and uncertainty-aware learning approaches capable of addressing these limitations. Bayesian deep learning and Monte Carlo-based techniques have emerged as promising solutions for improving prediction reliability and confidence estimation; however, their integration into end-to-end seismic monitoring systems remains relatively limited.

Overall, the reviewed literature suggests that future advances in intelligent seismic monitoring will depend on integrating several complementary research directions. These include the development of hybrid and attention-based architectures capable of jointly learning spatial and temporal dependencies, the incorporation of uncertainty quantification and interpretable artificial intelligence, and the design of transferable models that generalize across geographical regions, sensing technologies, and application domains. Furthermore, establishing standardized benchmark datasets and evaluation protocols will be essential for ensuring fair model comparison, improving reproducibility, and accelerating scientific progress. By synthesizing recent methodological developments, application domains, and existing research gaps, this review provides a comprehensive reference for researchers and practitioners and offers practical guidance for developing reliable, scalable, and trustworthy machine learning solutions for next-generation seismic monitoring systems.

## Figures and Tables

**Table 1:** Comparison of Existing Review Articles and the Present Study.

Feature	Existing Review	Present Review
Covers conventional seismic monitoring	Yes	Yes
Covers Distributed Acoustic Sensing (DAS)	Partial	Yes
Reviews both ML and DL methods	Yes	Yes
Systematic review following PRISMA	Limited	Yes
Focus on recent literature (2021–2025)	Partial	Yes
Compares datasets and application domains	Limited	Yes
Identifies research gaps	Partial	Yes
Discusses future research directions	Partial	Yes

**Table 2:** Comparison Between Conventional Seismometers and Distributed Acoustic Sensing (DAS) For Earthquake Magnitude Estimation.

Characteristics	Conventional Seismometer	DAS
Measurement	Ground velocity/acceleration	Strain/strain-rate
Sensor layout	Discrete stations	Continuous fiber
Spatial Resolution	Low Moderate	Very High
Magnitude Calibration	Established standards	Emerging Research
Data Volume	Moderate	Extremely Large
Real-Time Processing	Mature	Challenging
ML- Requirements	Conventional models	DAS-specific adaptation

**Table 3:** Search Strategy Used for Literature Retrieval.

Database	Search String
IEEE Xplore	(“earthquake monitoring” OR “seismic monitoring” OR “earthquake detection” OR “seismic event”) AND (“machine learning” OR “deep learning” OR “artificial intelligence” OR “neural network” OR CNN OR LSTM OR BiLSTM OR Transformer OR Bayesian) AND (“distributed acoustic sensing” OR DAS OR “fiber optic sensing”)
Scopus	TITLE-ABS-KEY ((“earthquake monitoring” OR “seismic monitoring” OR earthquake OR seismic) AND (“machine learning” OR “deep learning” OR “artificial intelligence” OR CNN OR LSTM OR BiLSTM OR Transformer OR Bayesian) AND (“distributed acoustic sensing” OR DAS OR “fiber optic sensing”))
Web of Science	TS = ((“earthquake monitoring” OR “seismic monitoring” OR earthquake OR seismic) AND (“machine learning” OR “deep learning” OR “artificial intelligence” OR “neural network” OR CNN OR LSTM OR BiLSTM OR Transformer OR Bayesian) AND (“distributed acoustic sensing” OR DAS OR “fiber optic sensing”))
Google Scholar	“earthquake monitoring” AND (“machine learning” OR “deep learning”) AND (“distributed acoustic sensing” OR DAS)

**Table 4:** Quality Assessment Criteria and Scoring Framework.

Criteria	Description	Score
Reproducibility	Availability of datasets, code, or sufficient methodological details	0/1
Model Transparency	Inclusion of explainability techniques and/or uncertainty quantification methods	0/1
Validation Strategy	Use of proper validation procedures (cross-validation, independent test set, Bayesian validation)	0/1
Performance Reporting	Clear and comprehensive reporting of evaluation metrics (e.g., Accuracy, F1-score, RMSE, and R^2^)	0/1
Total score	Sum of all satisfied criteria (maximum score = 4)	0–4
Quality Threshold	Studies satisfying ≥3 of the 4 evaluation criteria (≥70%) were included in the final qualitative synthesis	-

**Table 5:** Criteria of Inclusion and Exclusion.

Inclusion Criteria	Exclusion Criteria
Studies published between 2021–2025	Studies published outside the 2021–2025 publication period
Research focused on machine learning, deep learning, or artificial intelligence applications in earthquake monitoring, seismic analysis, or Distributed Acoustic Sensing (DAS)	Studies unrelated to earthquake monitoring, seismic analysis, geophysics, or DAS
Studies addressing seismic event detection, phase picking, earthquake classification, magnitude estimation, seismic hazard assessment, induced seismicity monitoring, or DAS-based monitoring	Studies focusing on non-seismic applications of machine learning
Peer-reviewed journal articles and conference papers	Editorials, book chapters, theses, patents, reports, and non-peer-reviewed publications
Studies reporting sufficient methodological details, datasets, model architectures, and evaluation metrics	Studies lacking methodological descriptions, datasets, or performance evaluation
Studies written in English	Non-English publications
Full-text articles available for detailed review	Studies without accessible full text
Studies using real seismic data, DAS data, benchmark datasets, or validated synthetic datasets	Studies containing only theoretical discussions without experimental validation
Studies reporting quantitative performance measures such as accuracy, precision, recall, F1-score, AUC, detection rate, localization error, or related evaluation metrics	Studies without quantitative results or validation evidence
Studies presenting original machine learning or deep learning methodologies, frameworks, or applications	Duplicate publications or substantially overlapping studies

**Table 6:** Summary of Literature Search and Screening Process Across Databases.

Databases	Initial Record	After Year Filter Version (2021–2025)	After Title/Abstract Screening	Final Selected Studies
IEEE Xplore	12,420	5876	51	16
WoS	32,369	12,789	135	28
Google Scholar	177,000	41,400	486	43
Scopus	30,434	4590	266	51
Total	252,223	64,655	938	138

**Table 7:** Summary of Related Work from SLR Studies.

Author(s) & Year	Learning Class	Algorithm(s)	Motivation	Contribution	Limitation	Application	Publisher	Publisher Rank
Li et al., 2025 [19]	Transfer/self-supervised learning	Self-supervised transfer learning-based deep neural network	Blind denoising of noisy DAS seismic signals to improve signal quality without requiring clean reference data.	Proposed a self-supervised transfer learning framework for DAS blind denoising, achieving superior denoising performance, improved adjacent-channel correlation, approximately 26 dB windowed SNR, and rapid inference for field applications.	Primarily addresses signal denoising and preprocessing rather than downstream seismic tasks such as event detection, phase picking, or earthquake classification.	DAS seismic signal enhancement and preprocessing for downstream seismic interpretation.	Photonic sensors (Springer)	Q2 Scopus CiteScore Quartile
Zhu et al., 2025 [20]	Supervised classification	MDLNet (CNN–BiGRU multimodal deep learning network)	Rapid seismic event discrimination and earthquake magnitude classification for earthquake early warning (EEW).	Developed MDLNet, a multimodal deep learning framework integrating time-domain, spectrum, and ground-motion features to improve seismic event discrimination and magnitude classification accuracy.	Validated only on the Japanese K-NET dataset and classifies earthquakes into three categories (microtremor, M < 5.5, and M ≥ 5.5), which may limit generalization to other regions and magnitude ranges.	Event detection and magnitude classification.	Geoscience Letters (Springer Nature	Q2
Hernández et al., 2022 [22]	Supervised learning	FC-ANN, CNN, and CNN+RNN	Develop an accurate deep learning framework for earthquake detection using Distributed Acoustic Sensing (DAS) measurements.	Demonstrated that deep learning models trained on broadband seismometer data can reliably detect earthquakes in DAS measurements, with CNN achieving 96.94% accuracy and CNN+RNN achieving 93.86% accuracy.	Performance slightly decreased when applied to DAS data because of differences between DAS and conventional seismometer waveforms and labeling uncertainties.	Earthquake detection using Distributed Acoustic Sensing (DAS)	Journal of Lightwave Technology (IEEE).	Q1
Wamriew et al., 2021 [23]	Supervised learning	Deep Neural Network (ResNet50-based Multitask Learning)	Automated detection, location, and velocity model inversion of DAS microseismic events for real-/semi-real-time monitoring.	Developed a multitask deep neural network capable of simultaneously detecting, locating microseismic events, and updating subsurface velocity models directly from DAS data with high accuracy.	Evaluated primarily using synthetic training data and limited field validation; broader evaluation on diverse geological settings is still required	DAS-based microseismic detection, event localization, and velocity model inversion	Sensors (MDPI)	Q1
Ding et al., 2025 [36]	Self-supervised learning	DASFormer (Transformer-based self-supervised pretraining framework)	Address the scarcity of labeled DAS data by learning transferable representations from large-scale unlabeled DAS datasets.	Proposed DASFormer, a self-supervised pretraining framework with a coarse-to-fine architecture that captures spatial-temporal correlations in DAS data and improves unsupervised earthquake detection and downstream tasks such as P/S phase picking.	Evaluated primarily on Ridgecrest DAS data; broader validation across diverse DAS deployments and geological settings is still required.	Earthquake detection, P/S phase picking, and DAS-based seismic monitoring	Visual Intelligence (Springer).	Q1
Li et al., 2024 [41]	Supervised Deep Learning	DynaPicker (Dynamic Convolutional Neural Network) + CREIME (CNN–RNN Magnitude Estimation)	Develop an automated deep learning workflow for real-time earthquake monitoring by integrating seismic phase picking, event detection, and magnitude estimation.	Proposed an integrated real-time earthquake monitoring framework combining DynaPicker for seismic phase detection and arrival-time picking with CREIME for magnitude estimation, and validated the workflow using the 2023 Kahramanmaraş Turkey aftershock sequence.	Validated primarily on conventional seismic waveform datasets rather than Distributed Acoustic Sensing (DAS) data; additional evaluation on diverse seismic regions is required.	Real-time earthquake monitoring, seismic phase picking, event detection, and magnitude estimation.	Solid Earth (Copernicus).	Q1
Hudson et al., 2025 [25]	Physics-informed/Hybrid Detection Framework	Physics-informed Back-migration Algorithm for Hybrid DAS–Seismometer Networks	Develop a robust earthquake detection and localization framework applicable to arbitrary DAS geometries and hybrid DAS–seismometer networks.	Developed a physics-informed back-migration framework that integrates DAS and conventional seismometer observations for earthquake detection and source localization across glacier, volcanic, and geothermal environments.	Computationally demanding and requires an accurate subsurface velocity model for back-migration.	Earthquake detection and source localization using hybrid DAS–seismometer networks.	Geophysical Journal International (Oxford University Press)	Q1

**Table 8:** Classification of References by Application.

Application Area	Sub-Area of Application	References
Earthquake Seismology, Earthquake Monitoring	Event detection, phase picking, catalog expansion, early warning systems, hypocenter location	[6,12,14,19,25,26,27,28,29,30].
Hydrocarbon Geoscience Exploration	Seismic subsurface characterization, fault and reservoir detection, lithofacies classification	[31,32,33,34,35,42,43,44,45].
Seismic Hazard and Risk Mitigation	Hazard analysis, earthquake forecasting, risk and hazard modeling	[36,46,47,48,49,50,51,52,53,54,55].
Seismic Monitoring of CO_2_ Storage and Subsurface Fluids	Induced seismicity detection, risk management in storage and injection operations	[56,57,58,59].
Engineering and Environmental Seismology	Earthquake engineering, structural response prediction, geotechnical hazard early warning	[37,38,60,61,62,63,64,65].
Volcanology and Geothermal Studies	Volcano-tectonic event detection, geothermal-induced seismicity prediction	[39,40,41,66].
Induced Seismicity and Anthropogenic Earthquake Studies	ML in human + industrial induced earthquakes	[67,68,69,70,71].

**Table 10:** Comparative Analysis of Machine Learning and Deep Learning Approaches for Seismic Monitoring Based on Performance Metrics, Strengths, Limitations, and Research Gaps. ↓: it means low uncertainty factor.

Method	Strengths	Weakness	Research Gaps	Performance Metrics (Typical)	References
CNN	Effective in spatial feature extraction; robust for seismic image and waveform analysis	Requires large labeled datasets; limited interpretability	Lack of cross-region validation; poor explainability	Accuracy: 85–95%; F1: 0.82–0.93 RMSE: 0.30–0.45; R^2^: 0.85–0.92	[1,7]
RNN	Captures temporal dependencies in seismic sequences	Vanishing gradient problem; slower training	Limited scalability for long sequences	Accuracy: 80–90%; F1: 0.78–0.88 RMSE: 0.35–0.50; R^2^: 0.78–0.88	[12,21]
LSTM	Handles long-term dependencies; suitable for time-series seismic data requires tuning	High computational cost;	Limited real-time deployment studies	Accuracy: 85–92%; RMSE: 0.25–0.40	[14,16]
Support Vector Machine (SVM)	Effective for small datasets; good generalization	Not suitable for large-scale data; sensitive to kernel choice	Limited performance on complex seismic signals	Accuracy: 75–88%; RMSE: 0.40–0.55; R^2^: 0.70–0.85	[40,72,73]
Random Forest (RF)	Robust to noise; handles non-linear relationships	Less effective for temporal dependencies	Limited use in real-time seismic monitoring	Accuracy: 80–90%; RMSE: 0.35–0.50; R^2^: 0.75–0.88	[18,23]
Gradient Boosting (XGBoost)	High predictive accuracy; handles feature interactions well	Risk of overfitting; requires parameter tuning	Limited application in DAS-based systems	Accuracy: 85–93%; RMSE: 0.28–0.42; R^2^: 0.85–0.92	[28,40]
K-Nearest Neighbors (KNN)	Simple and easy to implement; no training phase	High computational cost during inference	Poor scalability for large seismic datasets	Accuracy: 70–85%; RMSE: 0.45–0.60; R^2^: 0.65–0.80	[41,74]
Physics-Informed Neural Networks (PINNs)	Incorporates physical laws; improves interpretability	High computational cost; complex formulation	Limited large-scale validation	RMSE: 0.20–0.35; R^2^: 0.88–0.95	[64].
BiLSTM	Improved context learning (forward + backward)	Increased complexity and training time	Limited benchmarking across datasets	F1: 0.85–0.94; RMSE: 0.25–0.40; R^2^: 0.86–0.93	[49,70]
Transformer Models	Captures global dependencies; state-of-the-art performance	Requires large-scale training data; computationally expensive	Limited application in DAS real-time systems	Accuracy: 88–96%; F1: 0.86–0.95 RMSE: 0.22–0.35; R^2^: 0.88–0.94	[1,8,73]
Deep Belief Network (DBN)	Effective hierarchical feature learning	Complex training; less popular in recent studies	Limited modern applications in DAS data	Accuracy: 78–88%; RMSE: 0.35–0.50; R^2^: 0.75–0.87	[39,65]
Generative Adversarial Networks (GANs)	Useful for data augmentation and synthetic seismic data	Training instability; mode collapse issues	Limited validation in real seismic environments	RMSE: 0.25–0.40; R^2^: 0.82–0.90	[11,22,73]
CNN-LSTM Hybrid	Combines spatial + temporal learning	Complex architecture; difficult optimization	Lack of standardized evaluation datasets	Accuracy: 87–94%; F1: 0.84–0.92 RMSE: 0.20–0.35; R^2^: 0.90–0.96	[48,49]
Autoencoders	Effective for anomaly detection and denoising	Limited interpretability; reconstruction bias	Not widely validated for earthquake prediction	RMSE: 0.20–0.38; R^2^: 0.80–0.88	[19,27,73]
Self-Supervised Learning	Reduces need for labeled data; scalable	Requires large unlabeled datasets	Limited adoption in seismic applications	Accuracy: 82–90%; RMSE: 0.30–0.45; R^2^: 0.80–0.90	[51,52]
Graph Neural Networks (GNN)	Captures spatial relationships between seismic stations	Complex implementation; requires graph structure	Limited real-world deployment studies	Accuracy: 85–92%; RMSE: 0.25–0.40; R^2^: 0.85–0.93	[47,50]
Bayesian Models	Provides uncertainty quantification; interpretable	Computationally intensive; slower inference	Limited integration with deep learning models	RMSE: 0.28–0.42; R^2^: 0.82–0.90; Uncertainty ↓ 15–25%	[24,64]
DAS+ML Models	Real-time, high-resolution sensing capability	Data noise; massive data volume	Lack of standardized DAS datasets	Accuracy: 80–92%; RMSE: 0.30–0.48; R^2^: 0.80–0.89	[20,68]
Hybrid Ensemble Models	Improved robustness and accuracy	Increased model complexity; resource intensive	Limited generalization across regions	Accuracy: 90–97%; F1: 0.88–0.96; RMSE: 0.18–0.30; R^2^: 0.92–0.97	[18,25]

## Data Availability

Data can be made available upon request.

## Appendix A

**Table A1:** Glossary of abbreviations.

Abbreviation	Full Name	Abbreviation	Full Name
CNN	Convolution neural Network	BI-LSTM	Bi-directional Long short-term memory
LSTM	Long short-term memory	LR	Logistic/Linear regression
RF	Random forest	GNN	Graph Neural Network
AE	Auto encoder	VAE	Variational Autoencoder
SVM	Support vector machine	MC	Monte carlo
DAS	Distributed acoustic sensing	DT	Decision Tree
PCA	Principle component analysis	KNN	K-Nearest Neighbors
SOM	Self-Organizing Map	GA	Genetic Algorithm
GPS	Global Positioning System	WSN	Wireless Sensor Network
GNS	Global Navigation Satellite	InSAR	Interferometric Synthetic Aperture Radar
MEMS	Micro-Electro-Mechanical Sensor	UAV	Unmanned Aerial Vehicle
LIDAR	Light Detection and Ranging	USGS	United States Geological Survey
IRIS	Incorporated Research Institutions for Seismology	WoS	Web of science
STA/LTA	Short-Term Average/Long-Term Average	P/S WAVE	Primary/secondary waves
ML	Machine learning	AI	Artificial Intelligence
XG Boost	Extreme Gradient Boosting		

## Appendix B. Keyword Groups Used in Search

Seismic Terms

1. seismic data
2. seismic signal
3. earthquake
4. microseismic
5. seismic event
6. earthquake prediction

Distributed Acoustic Sensing Terms

1. distributed acoustic sensing
2. DAS
3. fiber optic sensing
4. optical fiber sensing

Machine Learning Terms

1. machine learning
2. deep learning
3. neural network
4. transformer
5. LSTM
6. BiLSTM
7. Bayesian inference
8. attention mechanism
